# Selenium intake is associated with gait speed in very old adults

**DOI:** 10.3389/fragi.2025.1473371

**Published:** 2025-07-02

**Authors:** Camila Dias Nascimento Rocha, Gilberto Simeone Henriques, Karine Aglio Vasconcelos, Lucca Ferreira Machado, Maria Aparecida Camargos Bicalho, Rodrigo Ribeiro dos Santos, Ann Kristine Jansen

**Affiliations:** ^1^ Postgraduate Program in Sciences Applied to Adult Healthcare, Universidade Federal de Minas Gerais, Belo Horizonte, Minas Gerais, Brazil; ^2^ Nutrition Department, Universidade Federal de Minas Gerais, Belo Horizonte, Minas Gerais, Brazil; ^3^ Postgraduate Program in Nutrition and Health, Universidade Federal de Minas Gerais, Belo Horizonte, Minas Gerais, Brazil; ^4^ Undergraduate Course in Nutrition, Universidade Federal de Minas Gerais, Belo Horizonte, Minas Gerais, Brazil; ^**5**^ Clinical Medicine Department, Universidade Federal de Minas Gerais, Belo Horizonte, Minas Gerais, Brazil

**Keywords:** longevity, healthy aging, selenium, biomarkers, minerals, physical performance, gait speed, skeletal muscle

## Abstract

**Introduction:**

Selenium seems to have protective effect on muscle function, contribute to healthy aging and longevity, however, in older adults this relationship has not been well studied. Objective: To evaluate the nutritional status of selenium in very old adults and its relationship with muscle strength, muscle mass and physical performance.

**Methods:**

A cross-sectional observational study investigating functionally independent individuals aged ≥80 years was conducted. Appendicular skeletal mass was determined by electrical bioimpedance, and physical performance and hand-grip strength were evaluated. Selenium intake and status in plasma and erythrocytes were evaluated. For statistical analysis. Poisson multivariate analysis was performed, and prevalence ratio used as a measure of effect.

**Results:**

A total of 72 older adults with a median age of 84 years were evaluated. Median Selenium intake was 71.58 mcg, and adequacy 73.5% overall, with a higher rate among men. In the total sample, 87.5% had optimal selenium plasma concentrations and no participants were deficient. All participants had adequate erythrocyte selenium levels. Gait speed was associated and correlated with selenium intake, even in the adjusted model The prevalence of low adequacy on the gait test was reduced by 3%–5% for every 1 mg increase in selenium consumption (PR 0.95; 95% CI: 0.93, 0.98).

**Conclusion:**

The gait speed results reinforce the hypothesis of an antioxidant role of selenium in muscle function. The very old adults studied demonstrated that homeostatic mechanisms control circulating selenium levels, highlighting the need for a specific reference value for the oldest-old population, besides the importance of analyzing blood markers associated with food intake and dietary patterns, since supplementation may prove iatrogenic.

## 1 Introduction

Aging is a biological process associated with increased oxidative stress, one of the most important mechanisms of cellular senescence (Martemucci et al., 2022). Although aging is inexorable, the way in which it occurs determines the vitality of the older person (Moraes, et al., 2016). Robust older adults have high intrinsic capacity and functional ability and are an example of healthy aging. Successful aging is a vector of the interaction among physical and mental health, economic independence, daily activities, social integration and family support (Depp and Jeste, 2006).

Studies on the aging process have identified mechanisms by which healthy lifestyle habits contribute to healthy aging, resulting in increased longevity (World Health Organization, 2015). In this context, nutrition is considered one of the hallmarks of aging (López-Otín et al., 2013). Several nutrients have been associated with increased quality of life and longevity (Ter Borg et al., 2016; Verlaan et al., 2017; Van Dronkelaar et al., 2023). However, studies evaluating the nutritional status of minerals are scarce (Van Dronkelaar et al., 2023; Seo et al., 2013; Ter Borg et al., 2016; Verlaan et al., 2017). Furthermore, there is a dearth of studies on the nutritional status of older adults aged 80 or over, the fastest growing strata of the population in Brazil and currently representing 14.6% of the Brazilian older population (Instituto Brasileiro de Geografia e Pesquisa, 2023). Studying this oldest-old group can help reveal new approaches for promoting healthy aging.

Selenium (Se) status is considered an important factor for maintaining health in older adults (Giovannini et al., 2018), where low plasma Se levels seem to be associated with a lower survival rate in this population (Alehagen et al., 2016; Giovannini et al., 2018), regardless of age or other clinical and functional parameters (Giovannini et al., 2018). The role of Se in aging and in the prevention of chronic diseases depends on its intake, differences in selenoprotein (Selp) genes, Selp expression, and the regulation of metabolic pathways, such as those related to the inflammatory response (Zhang et al., 2018; Ghashut et al., 2016).

Skeletal muscle is one of the main storage sites for Se (30%–45% of total pool) and deficiency in this mineral is believed to be associated with myopathy. Selp have a protective effect on muscle function (Castets et al., 2012; García-Esquinas et al., 2021), predominantly due to the action of glutathione peroxidase (GPx), which can neutralize reactive oxygen species (ROS) (Ighodaro and Akinloye, 2018). In addition, suboptimal levels of Selp also upregulate inflammatory cytokines, such as interleukin 6 (IL-6), causing muscle weakness, fatigue, pain and oxidative damage (Huang et al., 2012).

The association between selenium and sarcopenia, defined by loss of strength and muscle mass, is typically investigated in studies of individuals that are frail, present multiple comorbidities, are institutionalized, or in home care (Van Dronkelaar et al., 2023). However, few studies have explored the relationship between selenium and muscle health in older people exhibiting successful aging. Some studies have found that older adults with sarcopenia have lower selenium intake than those without sarcopenia (Ter Borg et al., 2016; Verlaan et al., 2017).

Thus, based on the hypothesis that the nutritional status of Se in very old adults with successful aging contributes to physical and muscular health, this study evaluated the nutritional status of Se in this oldest-old group and its relationship with muscle strength, muscle mass and physical performance, variables characterizing sarcopenia.

## 2 Materials and methods

### 2.1 Study design and setting

A cross-sectional, observational study of baseline data from a prospective, analytical open cohort study entitled “Mineral profile of older adults and relationship with healthy aging” was conducted. The study took place at the Multidisciplinary Care Clinic for Robust Elderly People and at Risk of Fragility of the Geriatrics and Gerontology Service of the Jenny de Andrade Faria Institute of the Hospital das Clínicas of the Federal University of Minas Gerais (HC/UFMG). The Geriatrics and Gerontology Service at the HC/UFMG is a Referral Center for Elderly Care in the State of Minas Gerais, Brazil.

The study was conducted in accordance with ethical guidelines and previously approved by the Ethics Committee of the same university under permit number 85566218.0.0000.5149. All participants signed a Free and Informed Consent Form.

### 2.2 Participants

The sample included all older adults evaluated between December 2018 and March 2023 who agreed to take part in the study. The inclusion criteria were individuals aged ≥80 years and functionally independent (robust) according to the multidimensional frailty model proposed by Moraes et al. (2016) in which the Functional Clinical Classification is systematized using the Visual analogue scale of frailty (VAS-Frailty). The use of this method is consistent with the International Classification of Functioning (ICF) of the World Health Organization (WHO), whose emphasis should be on functioning (Moraes et al., 2016). Centenarians were included in the study, regardless of the presence of frailty, as these individuals were considered models of successful aging (Borras et al., 2020).

**FIGURE 1:**
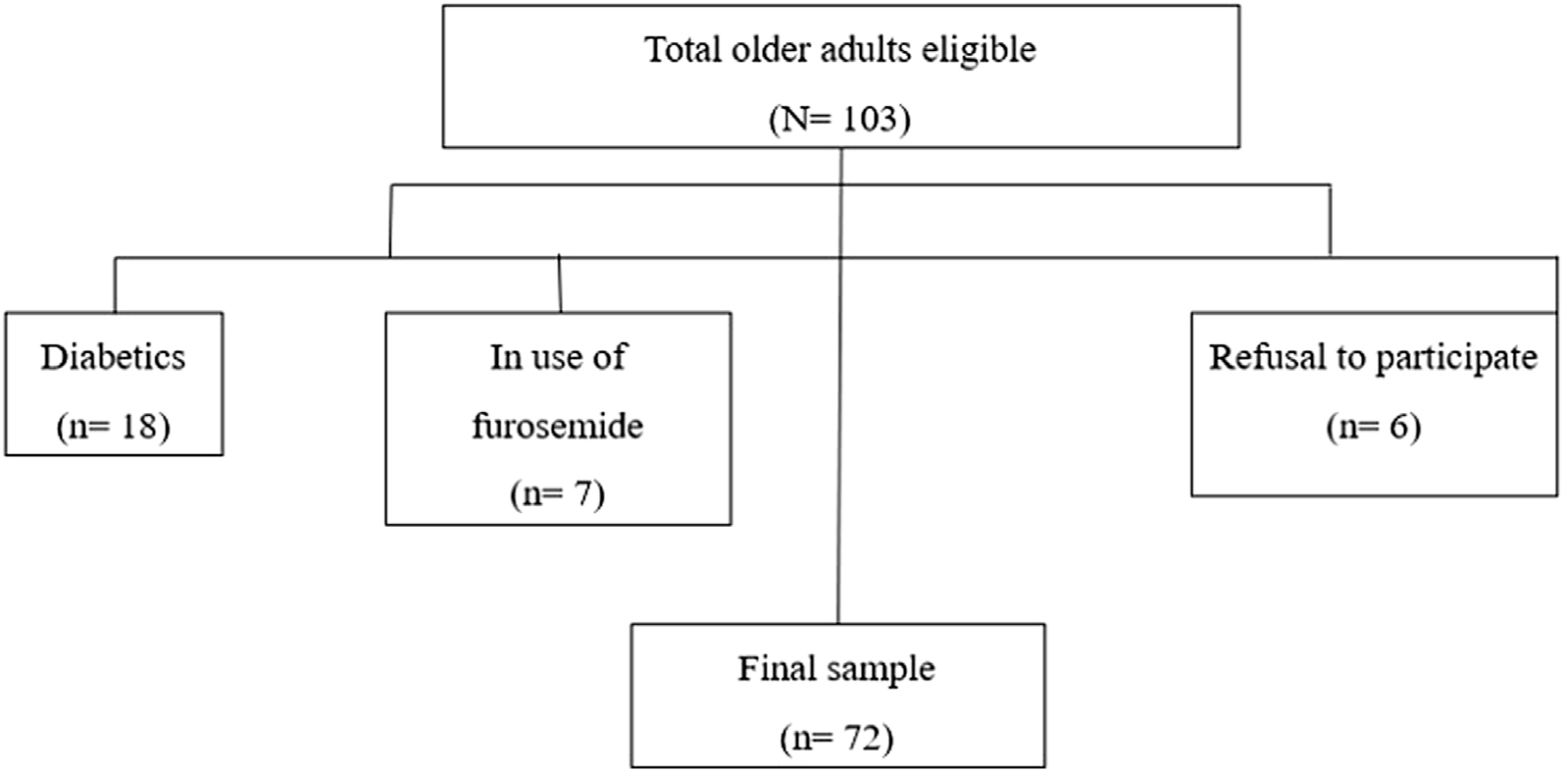
Sample selection based on eligibility criteria.

The exclusion criteria were institutionalized older adults, diabetics, those with chronic alcoholism and in use of furosemide, valproic acid or digitalis. Individuals who refused to take part in the study were also excluded. The sample selection process is presented in Figure 1.

### 2.3 Variables

The characteristics of the older adults determined were age, gender, years of education, per capita income, Pfeffer test score (Pfeffer et al., 1982), number of comorbidities, Charlson comorbidity index calculated (Charlson et al., 1987) by MD + Calc. (Quan et al., 2011), number of drugs, cognitive ability as measured by the Mini-Mental State Examination (MMSE) (Folstein et al., 1975), where 30 points was the maximum score and cut-off scores for no cognitive decline were defined as ≥13 points for subjects who were illiterate, ≥18 for those with low or medium education (elementary and secondary <8 years), and ≥26 for subjects with higher education (>8 years) (Bertolucci et al., 1994). Data for the variables physical activity and exercise (World Health Organization, 2018), nutritional status and physical capacity where also collected.

Nutritional status was evaluated by measuring weight on Filizola^®^ scales (PL 200 LED, Filizola^®^, São Paulo (SP) Brazil), and height using a stadiometer on the same equipment, according to the WHO recommendations (World Health Organization, 1995). Body Mass Index (BMI) was calculated, and the cut-off points used for classification were underweight (≤23 kg/m^2^), normal weight (>23 to <28 kg/m^2^), overweight (≥28 to <30 kg/m^2^) and obese (≥30 kg/m^2^) (Organización Panamericana de la Salud, 2002).

Body composition was assessed by an electrical bioimpedance test. A Biodynamics^®^ model 310 (Biodynamics corporation, United States) portable device with four poles was used, which emits an electric current of 800 microamps with 50 kHz intensity and frequency, respectively. The older adults were instructed to avoid strenuous physical exercise 12 h before the test, remove any metal objects and to fast for 8 h, according to the recommendations of Kyle et al. (2004).

The resistance (R) and reactance (Xc) data provided by the device were used to calculate fat free mass according to the equation of Kyle et al. (2001):

$$\mathrm{FFM}=-4.104+\left(0.518\;\times \mathrm{height}²/R\right)+\left(0.231\;\times \mathrm{weight}\right)+\left(0.130\;\times \;Xc\right)+\left(4.229\;\times \mathrm{sex}:\mathrm{men}=1,\mathrm{women}=0\right),\mathrm{where height is in centimeters}$$

Fat mass was obtained by subtracting body fat in kg from total body weight. Appendicular skeletal muscle mass (ASM) was calculated as proposed by Peniche et al. (2015):

ASM=−0.05376+0.2394 × height²/R+2.708 × sex+0.065 * weight,where height is in centimeters and sex for men=1 and women=0.

As recommended by the European sarcopenia consensus, ASM was divided by height^2^ to adjust for body size, and the cut-off point adopted was ≥7.0 kg/m^2^ and ≥5.5 kg/m^2^ for men and women, respectively (Gould et al., 2014).

Physical ability was evaluated using the Short Physical Performance Battery (SPPB) test validated for use in the Brazilian population (Nakano, 2007), including the assessment of static balance, lower limb muscle strength, and 4-m gait speed test. Good ability was defined as an SPPB score >8 (eight) points (Cruz-Jentoft et al., 2019). The 4-m gait speed test alone was also applied, with performance considered adequate for a mean value of >0.8 m/s (Cruz-Jentoft et al., 2019).

Hand-grip strength was measured using a Jamar^®^ dynamometer (BL5001, Lafayette, Indiana, United States) based on the highest of three measurements of both right and left hands interspersed, as recommended by Roberts et al. (2011). The cut-off points for adequacy were ≥27 kg and ≥16 kg for men and women, respectively (Dodds et al., 2014).

### 2.4 Selenium biomarkers

Plasma and erythrocyte Se concentrations were evaluated. Blood was collected in vacuum tubes with sodium citrate by a trained professional and participants were instructed to fast for 8 h. The samples were stored in a freezer at −20°C and diluted 1/30 (v/v) before analysis.

Inductively Coupled Plasma Optical Emission Spectrometry (ICP-OES), with an axial view configuration and V-Groove nebulizer connected to a hydride generation System (NaBH4) (Spectrometer 720 ICP-OES -, Varian Inc., California, United States), was used for mineral analysis. Samples were read at a wavelength of 203.985 nm, according to the methodology proposed by the Association of Analytical Chemists (AOAC) (Association of Analytical Chemists, 2012). Monoelemental stock solutions of Se of 1,000 mg.L-1 (Titrisol and Certipur - Merck, Germany) were used for the calibration curve. Samples of certified reference material, SeronormTM Oligoelement Serum L-1, and L-2 (Billingstad, Norway) were determined to validate the analytical measurements using ICP-OES.

The erythrocyte Se determinations were compared against the reference values of 0.18–0.55 μg/gHb (Vitoux, Arnaud, Chappuis, 1999). For plasma Se, optimal concentration was defined as ≥70.00 μg/L and deficient level as <45.00 μg/L (Perri et al., 2023).

### 2.5 Mineral intake

Food consumption was assessed using a food diary for 3 non-consecutive days including a weekend day, completed by the participant or their caregiver, recording all foods consumed and respective amounts in household measures, immediately after meals to avoid memory bias. Diaries were completed 1 week before blood. Questionnaires were returned to researchers for review of the food diaries, clearing up doubts and rectifying any additional details recorded incorrectly (World Health Organization, 1995).

The composition of calories, proteins, and Se was determined by calculating the mean of the 3 days of the food diary, including the amount per capita of sugar, salt, oil/fat, in addition to dietary supplements consumed. The nutritional composition assessment was performed according to the methodology proposed by the Brazilian Institute of Geography and Statistics (IBGE) (Instituto Brasileiro de Geografia e Pesquisa, 2011) in the “Brasil Nutri” program 2nd edition.

The daily caloric and protein intake was divided by body weight in Kg. Due to the lack of intrapersonal standard deviation values for Se, the percentage of older adults with adequate intake was calculated based on the adequate intake (AI) values for this mineral (Institute of Medicine, 2000).

### 2.6 Statistical analysis

The variables were tested for normality using the Kolmogorov-Smirnov test. Those variables with normal distribution were expressed as means and standard deviation (SD) and those with non-normal distribution as medians and interquartile range (IQR). Categorical variables were expressed as absolute frequencies and percentages.

The sample was dichotomized by gender for the analysis of characteristics, dietary intake, and Se biomarkers, and by adequacy of muscle strength, muscle mass and physical performance for the analysis of dietary intake of Se and its biomarkers.

Means were compared using Student’s t-test or the Mann-Whitney test for normal and non-normal distribution variables, respectively. The categorical variables were compared using Pearson’s chi-square test or Fisher’s exact test according to the ratio of expected frequencies lower than 5. Pearson or Spearman correlations were used to determine the relationships among selenium intake, biomarkers, muscle strength, muscle mass and physical performance.

Multivariate analysis was carried out using Poisson regression models with robust variance, where gait speed (adequate or low) was the dependent variable and the predictor variables with a p-value <20% (P < 0.20) was the explanatory variable, which were entered into the model using the backward method. The Hosmer-Lemeshow test was used to check the fit of the final model. Prevalence ratio (PR) with a 95% confidence interval (95% CI) was used as a measure of effect.

Data were analyzed using the Statistical Package for the Social Sciences program (SPSS version 21.0), except for the Poisson regression analysis, which was carried out using Stata software version 12.0 (Stata Corp, College Station, TX, United States). The level of statistical significance was set at p < 0.05.

## 3 Results

A sample of 72 older adults with a median age of 84 (82–87.75) years that included 3 centenarians and comprised 55.6% females was assessed. Median Pfeffer test score was zero, indicating good functioning (total independence for instrumental activities of daily living). Participant characteristics are shown in Table 1. Reasons for losses included problems completing the food diary, missing information on family income and difficulty carrying out the SPPB hand-grip strength tests.

**TABLE 1:** Characteristics of very old adults with successful aging, according to gender.

Variables	All participants (*N* = 72)	Male (*n* = 32)	Female (*n* = 40)	*p* -value
Age, years^a^	84 (82–87.75)	84 (81.25–86.75)	84 (82–90)	0.661†
Minimum and maximum age, years	80–108	80–108	80–101	
Octogenarians^b^	55 (76.4)	26 (81.3)	29 (72.5)	0.686*
Nonagenarians and centenarians^b^	17 (23.6)	6 (18.7)	11 (27.5)	0.225*
Education, years of formal study^a^	4 (2–8)	4 (3–6.75)	4 (3–6.60)	0.714†
*Per capita* income, USD^a^	238.8 (197.6–544.15)	233.19 (189.05–539.31)	208.37 (189.05–516.20)	0.784†
Number of comorbidities^a^	2 (1–3)	2 (1–2)	2 (1–2)	0.061†
Charlson comorbidity index^a^	5 (4–5)	5 (4–5)	5 (4–5)	0.751†
MMSE score^a^	26 (23–28)	26 (23.25–28.00)	26 (23.25–28.00)	0.386†
No cognitive decline^b^	62 (95.4)	27 (96.4)	35 (94.6)	1.00*
Number of drugs^a^	2 (2–4)	2 (2–4)	2 (2–4)	0.357†
Engages in physical activity^b^	63 (91.3)	28 (87.5)	35 (94.6)	0.405*
Engages in physical exercise^b^	37 (53.6)	20 (62.5)	17 (45.9)	0.227*
BMI, kg/m^2c^	26.3 ± 4.28	25.3 ± 4.16	27.05 ± 4.27	0.094#
BMI Classification^b^ Underweight Normal weight Overweight Obese	15 (20.8) 37 (51.4) 7 (9.7) 13 (18.1)	11 (34.7) 14 (43.7) 2 (6.2) 5 (15.6)	4 (10.0) 23 (57.5) 5 (12.5) 8 (20.0)	0.085*
Fat mass, kg^c^	22.11 ± 7.98	18.64 ± 7.49	24.74 ± 7.39	0.002#
% Fat mass^c^	34.26 ± 9.68	26.69 ± 6.62	39.98 ± 7.44	0.000#
Fat free mass, kg^a^	40.72 (34.77–46.70)	46.68 (44.32–55.99)	36.47 (32.40–39.46)	0.000†
ASM/h^2c^	6.35 ± 1.17	7.13 ± 0.84	5.74 ± 1.03	0.000#
ASM/h^2^ adequate^b^	38 (57.6)	15 (51.7)	23 (62.2)	0.457*
Gait speed, m/s^c^	1.03 ± 0.25	1.13 ± 0.23	0.95 ± 0.24	0.002#
Gait speed adequate^b^	54 (81.8)	27 (93.1)	27 (73)	0.053*
Grip strength, kg^c^	24.14 ± 8.51	30.83 ± 6.45	19.09 ± 4.51	0.000#
Grip strength adequate^b^	55 (80.9)	23 (76.7)	32 (84.2)	0.440*
SPPB score^a^	11 (10–12)	11 (10–12)	10 (9–12)	0.282†
Good physical performance^b^	54 (81.8)	25 (86.2)	29 (78.4)	0.527*

^a^ Median (IQR).

^b^ n (%).

^c^ Mean ± SD.

BMI, body mass index; cm, centimeters; g, grams; ASM, appendicular skeletal muscle mass; kg, kilograms; h, height; m, meters; MMSE, Mini-Mental State Examination; n, number; SD, standard deviation; s, seconds; SPPB, Short Physical Performance Battery. *p* -values derived from †Mann-Whitney U-test, *Pearson’s chi-square test or Fisher’s Exact test, #Student’s t-test for independent samples.

Median selenium intake was 71.58 mcg (52.45–90.60), with no gender difference, and overall adequacy was 73.5% with a higher rate among men (87.5% vs. 61.1%, P = 0.026) (Table 2). No difference in calorie, protein and selenium intake, or biomarkers was found between octogenarians, nonagenarians, and centenarians (data not shown).

**TABLE 2:** Calorie, protein and selenium intake and biomarkers in very old adults with successful aging, according to gender.

Variables	All participants (*N* = 72)	Gender		
		Male (*n* = 32)	Female (*n* = 40)	*p* -value
Calories, kcal^a^	1880.63 ± 550.43	2048.95 ± 449.58	1731.01 ± 593.31	0.016#
Kcal/kg, kcal^a^	30.03 ± 9.57	32.42 ± 9.16	27.90 ± 9.54	0.051#
Protein, g^a^	65.00 ± 20.56	70.78 ± 17.87	59.87 ± 21.64	0.028#
PTN/kg^b^	0.98 (0.79–1.3)	1.04 (0.90–1.32)	0.90 (0.75–1.25)	0.039†
Se intake, μg^b^	71.58 (52.45–90.60)	74.95 (62.25–87.83)	65.27 (48.90–96.71)	0.248†
Se intake adequate^c^	50 (73.5)	28 (87.5)	22 (61.1)	0.026*
Plasma Se, μg/L^b^	75.17 (71.60–76.9)	75.17 (73.28–76.91)	75.17 (70.67–76.93)	0.507†
Plasma Se adequate^c^	63 (87.5)	28 (87.5)	35 (87.5)	1.000*
Erythrocyte Se, μg/gHb^a^	0.37 ± 0.06	0.38 ± 0.07	0.37 ± 0.06	0.595#

^b^ Median (IQR).

^c^ n (%).

^a^ Mean ± SD.

Kcals, kilocalories; g, grams; kg, kilograms; Se, selenium; N, number; SD, standard deviation; µg: micrograms; L, liters; gHb, grams of hemoglobin; n, number. Reference Values (RV): plasma Se ≥ 70.00 μg/L ( Perri et al., 2023 ); erythrocyte Se = 0.18–0.55 μg/gHb ( Vitoux et al., 1999 ). *p* *-*values derived from †Mann-Whitney U-test, *Pearson’s chi-square test or Fisher’s Exact test, #Student’s t-test for independent samples.

Optimal plasma Se concentrations were found in 87.5% of the older adults and none had deficient Se concentrations. Erythrocyte Se was within reference values (RV) for all participants. No gender or age differences in selenium biomarkers were found (Table 2).

Results of evaluation of the association of muscle strength, muscle mass and physical performance with Se status revealed that only gait speed was associated with Se intake. Older adults with adequate gait speed consumed more selenium than those with low gait speed (74.54 mcg vs*.* 57.68 mcg) (Table 3).

**TABLE 3:** Association of muscle strength, muscle mass and physical performance with selenium intake and biomarkers in very old adults with successful aging.

Variables	Grip strength		
	Adequate (*n* = 55)	Low (*n* = 13)	*p* -value
Selenium intake, μg^a^	69.82 (57.63–94.44)	78.11 (55.98–91.90)	0.594†
Plasma Se, μg/L^a^	75.19 (71.08–77.08)	75.14 (72.12–76.30)	0.726†
Erythrocyte Se, μg/gHb^b^	0.37 ± 0.07	0.38 ± 0.04	0.845#

Variables	Physical performance (SPPB test)		
	Good performance (*n* = 55)	Low performance (*n* = 12)	*p*-value
Selenium intake, μg^a^	74.11 (59.78–95.66)	59.43 (48.80–80.21)	0.595†
Plasma Se, μg/L^a^	75.19 (71.27–77.05)	74.71 (72.89–76.44)	0.825†
Erythrocyte Se, μg/gHb^b^	0.37 ± 0.07	0.37 ± 0.06	0.987#

Variables	Gait speed		
	Adequate (*n* = 54)	Low (*n* = 12)	*p* -value
Selenium intake, μg^a^	74.54 (60.49–99.89)	57.68 (47.94–74.94)	0.034†
Plasma Se, μg/L^a^	75.19 (71.83–77.17)	74.32 (72.89–76.27)	0.589†
Erythrocyte Se, μg/gHb^b^	0.37 ± 0.07	0.37 ± 0.07	0.752#

Variables	Appendicular skeletal muscle mass/height^2^		
	Adequate (*n* = 38)	Low (*n* = 28)	*p* -value
Selenium intake, μg^a^	69.29 (49.45–93.91)	77.27 (60.84–97.14)	0.256†
Plasma Se, μg/L^a^	74.85 (70.23–77.02)	75.18 (73.17–76.94)	0.457†
Erythrocyte Se, μg/gHb^b^	0.36 ± 0.07	0.38 ± 0.05	0.336#

^a^ Median (IQR).

^b^ Mean ± SD.

Se, selenium; µg: micrograms; L, liters; gHb, grams of hemoglobin; n, number. *p-*values derived from †Mann-Whitney U-test, #Student’s t-test for independent samples.

No correlation was detected between Se intake and respective biomarkers. Plasma Se correlated weakly with erythrocyte Se (r = 0.287; P = 0.015). Regarding muscle strength, muscle mass and physical performance, only gait speed was correlated with Se intake (r = 0.324; P = 0.010) (data not shown).

According to regression analysis, the prevalence of low adequacy on the gait test reduced by 3%–5% for every 1 mg increase in selenium consumption, even after adjusting for gender, age, number of comorbidities, grip strength, engagement in physical exercise, and calorie and protein intakes (Table 4).

**TABLE 4:** Poisson Regression Analysis with robust variance for adequacy of gait speed among old adults with successful aging.

Adequacy of gait speed			
Variables	PR	95% CI	*p* -value
Model 1			
Selenium intake	0.97	0.95–0.99	0.003
Model 2			
Selenium intake	0.97	0.96–0.99	0.007
Model 3			
Selenium intake	0.95	0.93–0.98	0.001

Model 1: unadjusted, Model 2: adjusted for gender and age, Model 3: adjusted for gender, age, number of comorbidities, grip strength, engagement in physical exercise, and calorie and protein intakes.

PR, prevalence ratio; CI, Confidence Interval. Model fit: Goodness of fit = 0.99.

## 4 Discussion

The objective of the present study was to evaluate the selenium nutritional status in functionally independent very old adults and the relationship of this status with muscle strength, muscle mass and physical performance. Results showed high adequacy for Se intake, especially among men, and likewise for plasma Se concentration, besides very high adequacy for erythrocyte concentration. Only gait speed was associated with dietary Se intake, where participants with adequate walking speed consumed more of the mineral and every additional 1 mcg of Se intake was associated with a 3%–5% decrease in the prevalence of low gait speed.

Similarly, the Hertfordshire cohort study of 628 participants aged 63–73 years found that higher Se intake was associated with a shorter 3-m walk time in older women, with the authors suggesting this finding was due to the role of antioxidant nutrients in physical performance (Martin et al., 2011). Perri et al. (2020), in an analysis of data from the Newcastle 85+ study, found that older adults with low selenium intake had a 2.80 kg lower grip strength and a 2.3 s slower time on the Up-and-Go Test than older adults with high intake. However, selenium intake was inadequate in 53% of these participants compared with only 26.5% in the present study sample.

In relation to selenium intake, González et al. (2016) also evaluated consumption in Chilean very old adults, finding a mean of 84.5 (62–104) µg and 88.1 (56–109) µg per day in males and females, respectively, exceeding the median of 71.58 µg of Se per day (52.45–90.60) found in the present study. In the Newcastle 85+ Study, Se intake was 39.1 µg (Perri et al., 2023) while Jiménez-Redondo et al. (2014) found a Se intake of 62.3 ± 35.8 µg for females and 76.5 ± 29.5 µg for males in 83 older adults aged ≥80 years. For adequacy of Se consumption, the China national nutrition and health survey, involving 16,612 older adults, revealed Se intake inadequacy in 81.1% of those surveyed, with higher rates in participants aged ≥75 years compared to those aged 60–74 years (85.1% vs. 80.1%, respectively) (Liu et al., 2019). The National Diet and Nutrition Survey reported inadequate selenium intake in approximately 76% of women and 39% of men aged ≥75 years (Roberts et al., 2020). A Brazilian population study, the Household Budget Survey (POF 2008–2009), found a higher average consumption of 93.77 ± 2.27 µg per day in older adults (Instituto Brasileiro de Geografia e Pesquisa, 2011) compared to the present study, although the POF survey involved younger older adults.

With older age, there is a reduction in energy expenditure and concomitant intake, which affects the intake of micronutrients, although recommendations for mineral consumption do not differ with age (Institute of Medicine, 2000). However, the present study found no difference in dietary intake between octogenarians, nonagenarians, and centenarians, nor any correlation between age and selenium intake. The participants had a good intake of cereals, vegetables, legumes, beans, dairy products, and animal proteins, foods which contribute to selenium adequacy (González et al., 2006; Perri et al., 2023).

The disparities in results of the different studies might be attributed to the other characteristics of the older adults assessed, besides age. The diet–sarcopenia phenotype relationship may be affected by the inflammatory state of participants (Papadopoulou et al., 2023). Hence, older adults with multiple comorbidities and polypharmacy may experience changes in appetite, resulting in an impact on the way they eat (González et al., 2016). Moreover, underweight older adults possibly consume a lesser variety and quantity of foods than their normal-weight counterparts. The functional independence, number of comorbidities, low score on the Charlson index, good performance on the cognitive test, and low rate of polypharmacy observed in the present study are indicators of preserved health and, therefore, a lower degree of inflammation, a factor which may have contributed to the high adequacy in Se consumption identified. Also, 81.8% of the sample had good physical performance, 81.8% adequate gait speed and 80.9% adequate grip strength.

It is important to bear in mind that oldest old may have adapted to a lower Se supply and have physiological mechanisms to cope with this limited intake without this being detrimental to health (Perri et al., 2023).

The plasma and erythrocyte biomarkers of Se were found not to be associated or correlated with muscle strength, muscle mass and physical performance. In an observational study by Chen et al. (2014), serum Se was positively associated with muscle mass in older adult Indians (mean age 71.5 (±4.7) years) who were independent for basic and instrumental activities of daily living. In addition, a positive association of Se level with physical performance (Martin et al., 2011) and grip strength (Beck et al., 2007) was found, while a negative association with the prevalence of sarcopenia (Ter Borg et al., 2016; Verlaan et al., 2017) has also been reported. García-Esquinas et al. (2021) found plasma Se concentrations were inversely associated with physical function limitations (grip strength, performance, mobility, agility) in older adults, although the current investigation failed to confirm these associations.

Nevertheless, the literature is conflicting regarding the definition of reference values (RV) for the determination of Se in different compartments of the body and phases of the life cycle, potentially leading to inaccuracies in the determination of true nutritional status of Se (Combs, 2015). Furthermore, definitions in the literature for ‘adequate,’ ‘optimal’ and ‘deficient’ concentrations of Plasma Se concentration in older adults vary greatly (Lv et al., 2021; Perri et al., 2023). For this reason, the current study used raw data from Se markers when exploring the association of Se with muscle strength, muscle mass and physical performance. Giovannini et al. (2018), studying very old adults from an Italian community, found a higher median value of plasma Se (105.3 μg/L) than the present study (75.17 μg/L), yet the study by Perri et al. found a lower value (53.6 μg/L). Forte et al. (2014) observed a mean plasma Se concentration of 88.9 μg/L and 81.9 μg/L in 76 nonagenarians and 64 centenarians, respectively. Furthermore, some studies have found that plasma Se level reduces with age (Robberecht et al., 2019; García-Esqinas et al., 2021).

Plasma Se concentrations can be affected by several factors, such as inflammatory processes, low albumin levels, and circulating SelP (Stefanowicz et al., 2013). Thus, erythrocyte Se is a more reliable biomarker for examining the nutritional status of this mineral, remaining unchanged under certain conditions, such as the inflammatory response. In addition, studies have shown a strong correlation between selenium concentration in erythrocytes and the main metabolic pool of selenium in skeletal muscle (Stefanowicz et al., 2013). The absence of major comorbidities in the present study subjects suggests that low levels of oxidation require less plasma transfer of the mineral to the redox enzymes (Donadio et al., 2016). Furthermore, in chronic low-grade inflammation present in aging, SelP binds to the endothelium which leads to a drop in the plasma concentration of SelP and, consequently, of Se, since these proteins carry more than 70% of the Se in the plasma (Forceville et al., 2009). Erythrocytes have a long half-life and can contribute to the slow release of selenium into the circulating environment, leading to changes in nutritional status as measured by this biomarker (Stefanowicz et al., 2013), consistent with the current findings.

The present study has several strengths, such as the homogeneous sample in relation to age and functioning, plus its evaluation of plasma and erythrocyte biomarkers, together with the assessment of food intake records for a 3-day period. However, the study has some limitations, including the loss of some food diaries. Furthermore, given the cross-sectional design of the study, no cause–effect conclusions can be drawn.

## 5 Conclusion

The very old adults with successful aging with adequate gait speed had higher selenium intake than those with low gait speed, confirming the hypothesized role of this antioxidant mineral in muscle function. In addition, this group of older adults demonstrated that homeostatic mechanisms preserve circulating Se levels, temporarily compensating for low dietary intake.

The present study of individuals with more advanced chronological age highlights the need for a specific RV for this population. New approached for promoting healthy aging and preventing sarcopenia may be revealed by studying mineral nutrition status in this group.

Expanding the data on blood markers associated with the analysis of both food intake and dietary patterns is of fundamental importance in the population analyzed. Although the needs and recommendations for selenium have not yet been established for older adults, the study of the oldest old can further understanding on the true need for the mineral, its use in biological systems, the risk of deficiency, and decisions regarding its supplementation, a practice which may prove iatrogenic.

Although this question is beyond the scope of the present cross-sectional study, further investigations in an area with scant information on very old adults should be encouraged.

## Data Availability

The original contributions presented in the study are included in the article/supplementary material, further inquiries can be directed to the corresponding author.
